# Room temperature bioproduction, isolation and anti-microbial properties of stable elemental copper nanoparticles

**DOI:** 10.1016/j.nbt.2017.10.002

**Published:** 2018-01-25

**Authors:** Nikolaos Pantidos, Matthew C. Edmundson, Louise Horsfall

**Affiliations:** School of Biological Sciences and Centre for Science at Extreme Conditions, University of Edinburgh, The King’s Buildings, Alexander Crum Brown Road, Roger Land Building, Edinburgh, EH9 3FF, United Kingdom

**Keywords:** Elemental copper nanoparticles, Biosynthesis, Anti-microbial

## Abstract

In nanoparticle production there are a number of important considerations that must be made. Producing nanoparticles of uniform size and shape is vital, but no less important is ensuring the production process is as efficient as possible in time, cost and energy. Traditional chemical and physical methods of nanoparticle production often involve high temperatures and pressures, as well as the use of toxic substrates; in contrast the bioproduction of nanoparticles is greener and requires a smaller input of energy resources. Here we outline a method for the straightforward bioproduction of stable, uniform elemental (zero-valent) copper nanoparticles at room temperature, and demonstrate how their size and shape can be modified by subsequent pH manipulation. We also highlight a potential application for these biogenic copper nanoparticles by demonstrating their potential to inhibit bacterial growth.

## Highlights

• Synthesis and characterization of elemental, zero-valent copper nanoparticles.
• Copper nanoparticles synthesised by *M. psychrotolerans* are stable for up to 3 months.
• Growth inhibiting properties of copper nanoparticles.

## Graphical abstract

**Figure:**
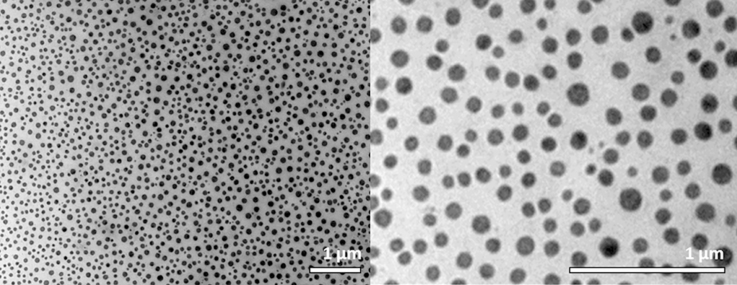

## Introduction

Metallic nanoparticles are widely used in many industrial and medical applications [1], [2], [3]. Some forms such as elemental (zero-valent) copper nanoparticles are known to be anti-microbial [4], whilst others such as zinc oxide nanoparticles have catalytic properties useful in the production of methanol [5]. Currently, silver nanoparticles are used in the new field of nanoelectronics to produce conductive inks to print electric circuits for inexpensive electrical items such as radio-frequency identification tags [6]. Copper nanowires have also been shown to display useful electrical properties [7] and it may therefore be possible to replace silver with copper nanoparticles in these applications, thereby reducing the cost of production and of the raw material. The demand for nanoparticles of many different metals is currently being met using chemical or physical synthesis techniques [8]. These methods are often energy-intensive, requiring high temperatures and pressures [9], [10], [11] and the need for expensive starting materials can also increase the cost of synthesis; in addition, many processes require the use of hazardous chemicals [12]. Copper nanoparticles are difficult to produce using traditional physical and chemical methods [13]; it is possible to use hydrazine as a reducing agent in copper nanoparticle synthesis [14], but this compound is highly toxic, while alternative methods call for high temperature (80–200 °C) steps [15], [16]. Furthermore, these chemically or physically synthesised copper nanoparticles are unstable, being readily oxidised in air and aqueous solutions [14], [17].

The field of nanomaterial production using biological entities has come a long way in just over two decades. The number of studies in this area has been on the rise as the societal need to find alternative eco-friendly methods for nanomaterial production is becoming stronger over time [18], [19]. Newer, ‘greener’ production techniques are being explored in the field of biogenic nanoparticle production [12], [20]. Biological processes usually occur in aqueous solutions at, or near, room temperature and standard atmospheric pressure, reducing energy costs and the need for hazardous solvents and reagents. Additionally, with a suitable biological reaction pathway in place, standard low-cost bulk chemicals can be used as the raw materials of nanoparticle production, such as copper sulphate in the case of copper nanoparticles [13]. The starting material does not have to be pure, and could even be copper contaminated waste, soil or water; using bacteria to remediate such waste by converting the copper contaminants into useful nanoparticles would add value to the bioremediation process. Similarly, sand and rice husks have been used as starting materials for the production of silica nanoparticles using *Fusarium oxysporum* through bioleaching [21], [22], while a separate study showed the specificity advantages of using biological processes, through selective bioleaching of silica from sand [23]. A number of organisms naturally produce metal nanoparticles [24], [25], [26], [27] and this provides a ready-made platform to investigate bioproduction. As well as these advantages bioproduction may also help to stabilise nanoparticles; proteins are able to bind to the nanoparticles, preventing their oxidation when exposed to air [28]. This stabilisation is important as it maintains the optical and anti-microbial properties associated with the elemental form of copper nanoparticles.

To pursue the goal of producing useful biogenic copper nanoparticles we have looked to the bacterial genus *Morganella*, which contains species able to synthesise several different metallic nanoparticles. *M. morganii* has been reported to synthesise nanoparticles of both silver and copper [13], [29], but for this work we chose *Morganella psychrotolerans* as it produces one of the highest observed yields of silver nanoparticles of all the various *Morganella* species tested to date [30], and here we have demonstrated that it is also able to produce elemental copper nanoparticles. In addition, *M. psychrotolerans* unlike *M. morganii* is a class 1 organism, meaning all laboratory work and any future applications can be done easily and safely. Its ability to grow in both aerobic and anaerobic conditions is also an advantage as it allows the study of various nanoparticle synthesis conditions. Furthermore, making this species attractive for nanoparticle bioproduction is its preferred growth temperature of 20 °C. This low-temperature growth reduces the energy costs of nanoparticle synthesis and it has been reported that sub-37 °C temperatures may aid the formation of nanoparticles [29].

Regardless of the intended use for the nanoparticles, their purity and homogeneity of size and shape are vitally important; only by having consistent shape and size both within and between batches can consistency and reliability be maintained. By manipulating pH previously in other organisms, it was possible to affect nanoparticle morphology [31]. We have used pH in a similar way to alter the morphology and homogeneity of nanoparticles produced by *M. psychrotolerans*, producing copper nanoparticles with a variety of sizes and shapes, as well as populations of highly uniform nanoparticles. In addition to being able to synthesise nanoparticles, a purification technique was developed to separate the nanoparticles from the bacteria and any other components present in the growth medium.

Anti-microbial resistance has become a great challenge in recent years and drug options to treat patients are becoming increasingly limited [32]. The continuing rise of this resistance in recent years has led to efforts to develop new types of anti-microbials. Copper nanoparticles have been shown to exhibit anti-microbial effects against *E. coli* cultures [33]. Therefore, we are investigating our biogenic elemental copper nanoparticles for their potential use as anti-microbial agents.

This study sets out a simple method for the green bioproduction, purification, subsequent manipulation, and anti-microbial application of biogenic elemental copper nanoparticles. We also propose that this method might easily be applied to other metallic nanoparticles.

## Materials and methods

### *Morganella psychrotolerans* cultures

*M. psychrotolerans* was obtained from the German collection of microorganisms and cell cultures (DSMZ). Cells were grown in low-salt LB medium (5 g L^−1^ yeast extract, 10 g L^−1^ tryptone). For aerobic nanoparticle synthesis, cells were incubated at 20 °C in a shaking incubator (200 rpm) for 24 h. For anaerobic nanoparticle synthesis, low-salt LB medium was degassed and then *M. psychrotolerans* was incubated at 30 °C in an anaerobic cabinet under 10% CO2, 10% Hydrogen, Nitrogen Anaerobic Mixture at 200 rpm for 24 h. To produce copper nanoparticles copper sulphate (Sigma-Aldrich) was added to the growth medium to a final concentration of 5 mM. After this the cultures were incubated for a further 24 h, aerobically or anaerobically as required. After a total incubation of 48 h the cultures were centrifuged at 4000 rpm (3200*g*) for 10 m to pellet the cells. The supernatant was removed and filtered through a 0.22 μm filter to take out any remaining cells, with copper nanoparticles present in the filtered supernatant.

### UV wavelength measurements

*M. psychrotolerans* was grown as before for 24 h (20 °C, 200 rpm) aerobically in biological triplicate. Copper sulphate was added (final concentration 5 mM) and the culture briefly mixed before having 1 ml removed and returning the remaining culture to the incubator. For the negative control cultures no copper was added but all other steps are the same. This 1 ml sample was spun at 8000 rpm (6800 g) for 1 m in a benchtop centrifuge and the supernatant added to a disposable UV cuvette. A full spectrum scan was performed for T = 0 between the wavelengths 200 nm–800 nm using a spectrophotometer (UVmini-1240, Shimadzu). Further samples were taken, centrifuged and scanned at T = 1 h, 2 h, 3 h, 4 h, 6 h, 8 h, 12 h and 24 h.

### Nanoparticle formation in buffer

*M. psychrotolerans* cells were incubated in 40 ml of low-salt LB medium and taken from the incubator after 24 h. They were then centrifuged at 4000 rpm (3200*g*) for 10 m. The pellets were resuspended in 50 mM Tris–HCl pH 7.6 and centrifuged at 4000 rpm (1700*g*) for 3 m. This wash step was repeated twice more. After the final wash the cell pellets were resuspended in 10 ml Tris–HCl, pH 7.6; to achieve nanoparticle formation copper sulphate was added to the buffer at this stage (final concentration 5 mM). The buffered cells were then incubated for a further 24 h (20 °C, 200 rpm). The samples were then centrifuged and filtered in the same way as those incubated solely in low-salt LB.

### Purification by ultracentrifugation

To a Beckman polyallomer ultracentrifuge tube (14 × 95 mm) was added 15 ml of filtered supernatant (prepared from *M. psychrotolerans* low-salt LB cultures) or 10 ml of filtered buffer (prepared from *M. psychrotolerans* incubated in Tris–HCl pH 7.6) topped up to 15 ml using Tris–HCl for each sample. The samples were centrifuged in a Beckman XL-100 ultracentrifuge using the SW40-Ti rotor at 39,000 rpm (270,000*g*) for the chosen length of time for each experiment (30 m, 45 m, 3 h and 16 h). Following centrifugation the supernatants were carefully removed and retained, and the pellets were resuspended in buffer, either MOPS (pH 6.5 or 7.5) or Tris–HCL (pH 6.5), and incubated for 24 h at 4 °C. Suitable controls were also performed, including *M. psychrotolerans* grown in the absence of copper and non-inoculated media, both with and without copper.

### Purification by stirred cells

A 50 ml culture of *M. psychrotolerans* grown to stationary phase overnight was transferred into a stirred cell (50 ml Amicon Stirred Cell, Millipore). The culture was filtered using a cut-off membrane filter of 220 nm to separate the bacterial cells from the nanoparticles. Nitrogen gas was pumped into the cell to create a positive pressure for filtration at 5 bar. The nanoparticles were then washed and concentrated by filtering using a cut-off membrane of 3 kDa, trapping them in the suspension above the filter. After concentration, the nanoparticles were ready for further use.

### ICP-OES

Copper nanoparticle suspensions were measured using Inductively Coupled Plasma–Optical Emission Spectrometry (ICP-OES). A minimum of 3 ml of each sample was collected, nitrified by adding 5% final nitric acid, sonicated for 15 m and copper concentrations measured using a Perkin Elmer Optima 5300 DV ICP-OES, using an Argon plasma torch.

### Transmission electron microscopy imaging

Following incubation in buffer, each sample was drop-cast onto a copper grid. Excess sample was removed by blotting paper and the grid was left to air-dry. Samples containing nanoparticles were briefly centrifuged (30 s, 1700*g*) to pellet any large clumps of nanoparticles as these could damage the grids when in the electron beam. The TEM used was a Philips CM120 transmission electron microscope at 70 kV. Images were taken on a Gatan Orius CCD camera.

### Anti-microbial assays

Copper sulphate, non-biogenic (Sigma-Aldrich, catalogue number 774103) and biologically synthesised copper nanoparticles were tested for their anti-microbial properties against *Bacillus subtilis*, as well as against the producer strain *M. psychrotolerans* to investigate any possible toxic effects that may affect its growth. Concentrations of 126.0, 63.0, 31.5, 15.8 and 0 ppm of biogenic copper nanoparticles were prepared in low-salt LB medium (for *M. psychrotolerans*) and LB medium (*for B. subtilis*) in 96-well plates. The wells were inoculated with *B. subtilis* or *M. psychrotolerans* and the plates were subsequently incubated at 30 °C (*B. subtilis*) or 20 °C (*M. psychrotolerans*) for 24 h with vigorous shaking. Readings were taken every 2 h at O.D._600_.

### Fast Fourier transform images

For FFT images, the samples were prepared similarly to the TEM samples. The sample was imaged on a JEOL JEM 2011 HRTEM under an accelerated voltage of 200 kV.

## Results and discussion

**Fig. 1:**
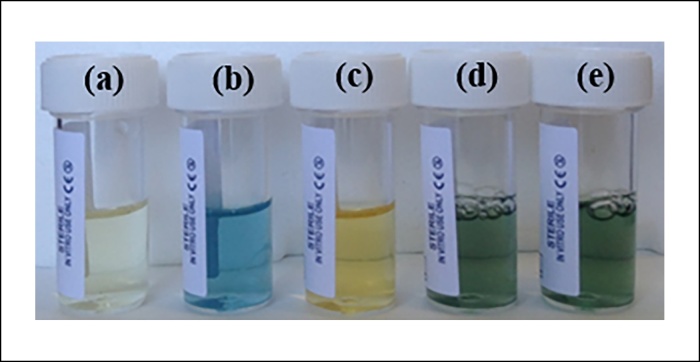
Colour changes occuring during *M. psychrotolerans* incubation with and without copper sulphate. From left to right: LB medium; LB medium with 5 mM copper sulphate; ‘spent’ LB medium after 48 h incubation; medium from *M. psychrotolerans* culture containing 5 mM copper; spent LB medium with 5 mM copper sulphate added after *M. psychrotolerans* cells have been removed.

After incubating *M. psychrotolerans* in low-salt LB containing 5 mM copper sulphate (CuSO_4_) a change in colour of the media is observed, going from blue immediately following CuSO_4_ addition (Fig. 1b) to green after 24 h incubation (Fig. 1d). This has previously been reported as an indication of the formation of copper nanoparticles [13]. but is in fact due to changes in the colour of the medium itself; there is a clear colour difference between fresh LB (Fig. 1a) and spent LB i.e. after incubation with *M. psychrotolerans* (Fig. 1c), and it is this more intense yellow colour mixing with the blue of the copper sulphate (CuSO_4_) that causes the medium to turn green. Indeed filtered media that has been used to grow *M. psychrotolerans* in the absence of CuSO_4_ will turn green upon the immediate addition of CuSO_4_ (Fig. 1e).

**Fig. 2:**
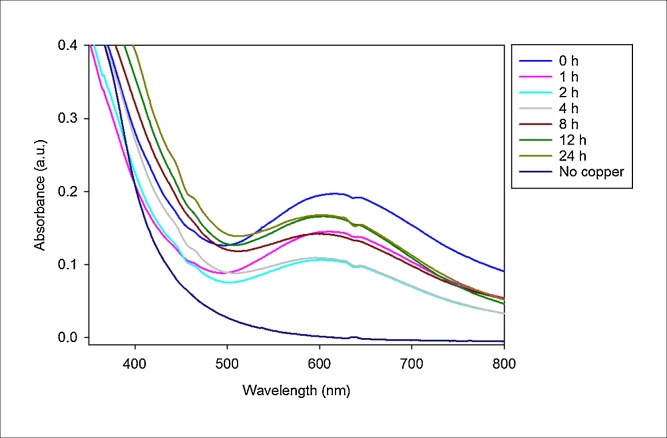
Graph showing absorbance readings in a spectrum scan between the wavelengths 200 nm and 800 nm following the formation of copper nanoparticles. Scans were performed at T = 0, 1 h, 2 h, 4 h, 8 h, 12 h and 24 h.

*M. psychrotolerans* produces copper nanoparticles when incubated with CuSO_4_ in low-salt LB, and their formation can be followed using spectrophotometry (Fig. 2). Cultures of 350 ml of *M. psychrotolerans* were grown and concentrated down for the experiment; using smaller cultures did not produce any noticeable change in absorbance. Following addition of CuSO_4_ at T = 0 there is an immediate jump in absorbance of the supernatant between 600 nm and 620 nm, caused by the copper ions. This absorbance sharply decreases during the first two hours, due to the cells absorbing the copper ions. For the next ten hours the absorbance of the samples increases. This increase in absorbance is due to the formation of copper nanoparticles accumulating over time in the supernatant. Between 2 and 4 h, sufficient nanoparticles have accumulated to counteract the decrease in absorbance due to the uptake of copper ions, and this is observed as an increase in absorbance reading.

X-ray Photoelectron Spectroscopy (XPS) was performed on copper nanoparticles prepared over a period of six months to analyze their stability. The nanoparticles showed high stability, being observed to remain elemental in nature up to 3 months post-production. Thereafter XPS data indicated that oxidation had occurred, with Cu_2_O being observed after 3 months of storage at 4 °C (Fig. A1).

**Fig. 3:**
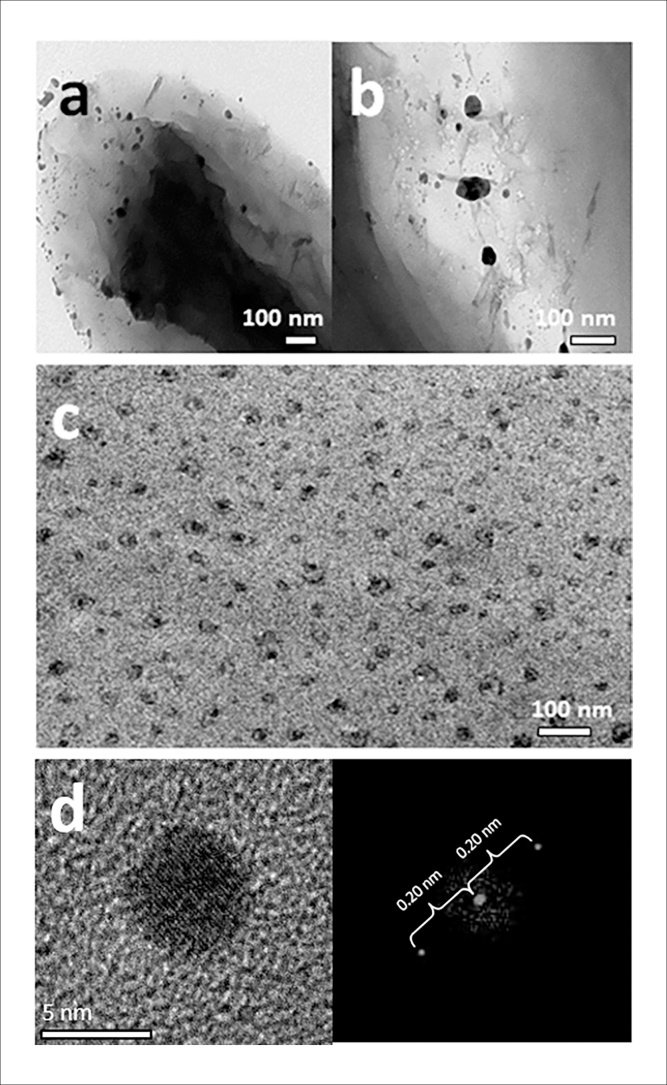
Images of copper nanoparticles produced by *M. psychrotolerans*. (a, b) TEM images of *M. psychrotolerans* cells with copper nanoparticles on the cell membranes. Nanoparticles range from 4 to 60 nm. (c) Copper nanoparticles produced by *M. psychrotolerans* with bacterial cells removed. (d) TEM image of a copper nanoparticle (left) and the FFT image showing the d-spacing measurement of that nanoparticle (right). The d-spacing value is 0.20 nm, the value for pure copper.

The nanoparticles were observed using transmission electron microscopy (TEM) (Fig. 3a–c). They were irregular in size and shape, with a diameter from 4 nm up to 60 nm in some cases. Selected area electron diffraction Fast Fourier Transform (FFT) pattern analysis of the nanoparticles suggested that they were composed of pure, elemental copper (Fig. 3d) with diffraction spacings of 0.20 nm [13].

To optimise nanoparticle production, various buffers were investigated for their pH effects on nanoparticle formation by *M. psychrotolerans*. Cells were grown in low-salt LB for 24 h, washed and transferred into buffers at pH 6.5 and 7.5 (MOPS) and pH 7.6 (Tris HCl). After incubation of the cells in these buffers supplemented with CuSO_4_, only the cells incubated in Tris–HCl remained viable, but no nanoparticle synthesis was observed. We therefore reason that there is a nutrient requirement for the successful formation of copper nanoparticles by *M. psychrotolerans*, and this is fulfilled by the growth medium, with optimal nanoparticle formation taking place in low-salt LB. Any manipulation of nanoparticle morphology using pH must therefore be done post-production.

**Fig. 4:**
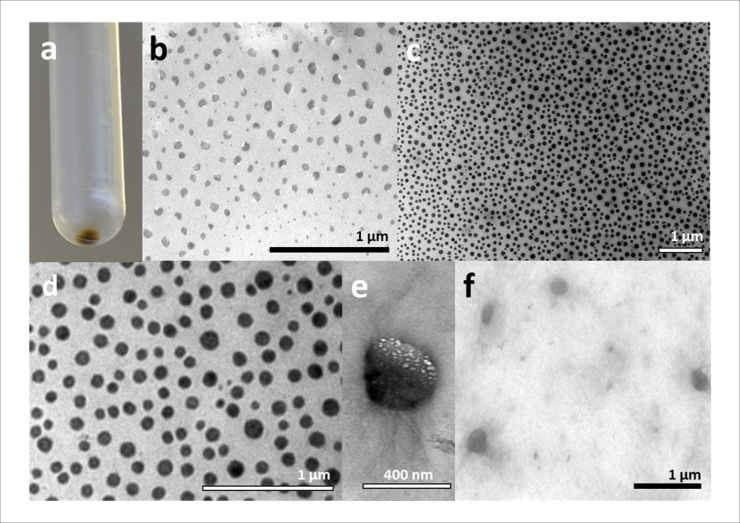
Images of copper nanoparticles following purification and nanoparticle manipulation by pH. (a) Copper nanoparticle pellet formed after over-night ultracentrifugation. (b) Supernatant containing copper nanoparticles following ultracentrifugation for 30 min. Debris from the LB medium has largely been removed; the nanoparticles retain the irregular shape and sizes of the nanoparticles produced by *M. psychrotolerans* (Fig. 3a, c). (c) Copper nanoparticles following 24 h of incubation in MOPS buffer pH 7.5. The nanoparticles are now more uniform in shape, and most are around 100 nm. (d) A close-up of a section from (c). (e, f) Copper nanoparticles following 24 h of incubation in MOPS buffer pH 6.5. The nanoparticles are irregular in size and shape but some are bigger than the nanoparticles found before incubation in buffer, up to 400 nm in some instances (e).

Ultracentrifugation, either with or without a chemical gradient [34], can be employed as a means to purify nanoparticles. Here we used ultracentrifugation to concentrate the biogenic copper nanoparticles into a pellet (Fig. 4a), with few nanoparticles pelleted after 30 min but the majority pelleted following 16 h ultracentrifugation. Nanoparticles recovered by ultracentrifugation displayed the same morphology and size range as those taken directly from the *M. psychrotolerans* culture supernatant (data not shown). Centrifugation can therefore be used as a means to remove the debris associated with the medium. By performing a shorter centrifugation of 30 min, the majority of contaminants were removed while not significantly reducing the yield of copper nanoparticles (Fig. 4b). Extended ultracentrifugation times were used to isolate and concentrate the majority of the nanoparticles.

These nanoparticles can be considered as ‘seeds’; they are the “raw” form produced by the bacteria and further processing and manipulation yields other structures. Resuspending the nanoparticle pellet in buffers of different pHs and incubation for a period of 24 h produced marked differences in nanoparticle morphology when viewed using TEM. At pH 7.5 the nanoparticles produced were larger compared to the seed nanoparticles and their sizes were also more uniform, with the majority having a diameter of approximately 100 nm (Fig. 4c, d). At the same time the nanoparticles showed a significant similarity in shape, becoming more rounded than the seed nanoparticles. Incubation at pH 7.5 is therefore an ideal way of converting the purified copper nanoparticle seeds into uniform, non-aggregated nanoparticles, allowing a thorough assessment of their properties.

At pH6.5, the nanoparticles retained the same morphology as at pH7.5, appearing in similar numbers and densities. However, some of the seed nanoparticles merged and increased in size, with diameters up to 200 nm (Fig. 4g, h), and would be more correctly termed microparticles. While it is possible that these may have been aggregates of nanoparticles that have failed to resuspend, they were not observed in any other sample at any other pH. These large particles were confirmed to be copper by energy-dispersive X-ray spectroscopy (EDX) analysis (data not shown), and could have properties very different to those of the smaller, homogenous nanoparticles.

**Fig. 5:**
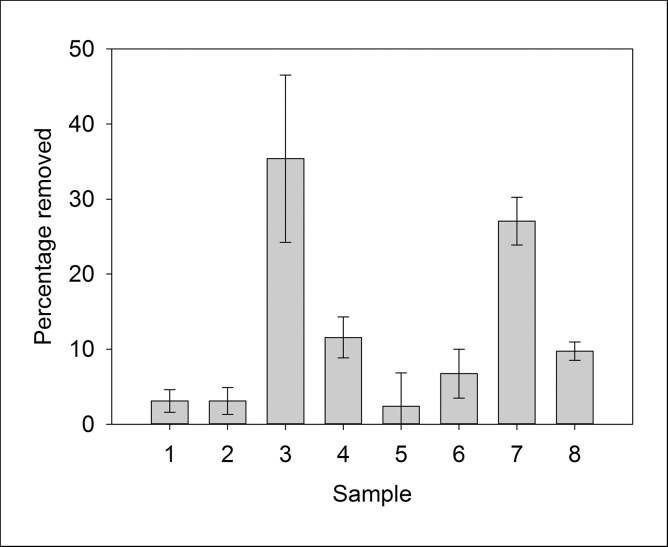
Bar chart showing concentrations of Cu^2+^ converted into copper nanoparticles using *M. psychrotolerans* in various conditions. 1) Aerobic shaking 1 mM, 2) Aerobic static 1 mM, 3) Anaerobic shaking 1 mM, 4) Anaerobic static 1 mM, 5) Aerobic shaking 0.5 mM, 6) Aerobic static 0.5 mM, 7) Anaerobic shaking 0.5 mM, 8) Anaerobic static 0.5 mM.

Copper removal was measured by ICP-OES. Using the original method for removing copper, only a small amount of nanoparticles was produced and collected (Fig. 5). To investigate whether yield can be increased, a range of incubation conditions for nanoparticle production were tested. Cultures were grown aerobically or anaerobically under shaking or non-shaking conditions. Using ICP-OES, nanoparticle yields were compared between the original aerobic synthesis conditions as well as anaerobic conditions (Fig. 5). The highest yield was observed to be synthesised under vigorous shaking anaerobic conditions, possibly due to the absence of oxygen affecting the nanoparticles during aerobic shaking conditions. By means of this method we were able to convert up to 32% of the copper ions present in the sample into nanoparticles. Using stirring cells it was also possible to extract them from the culture and concentrate them for further use.

**Fig. 6:**
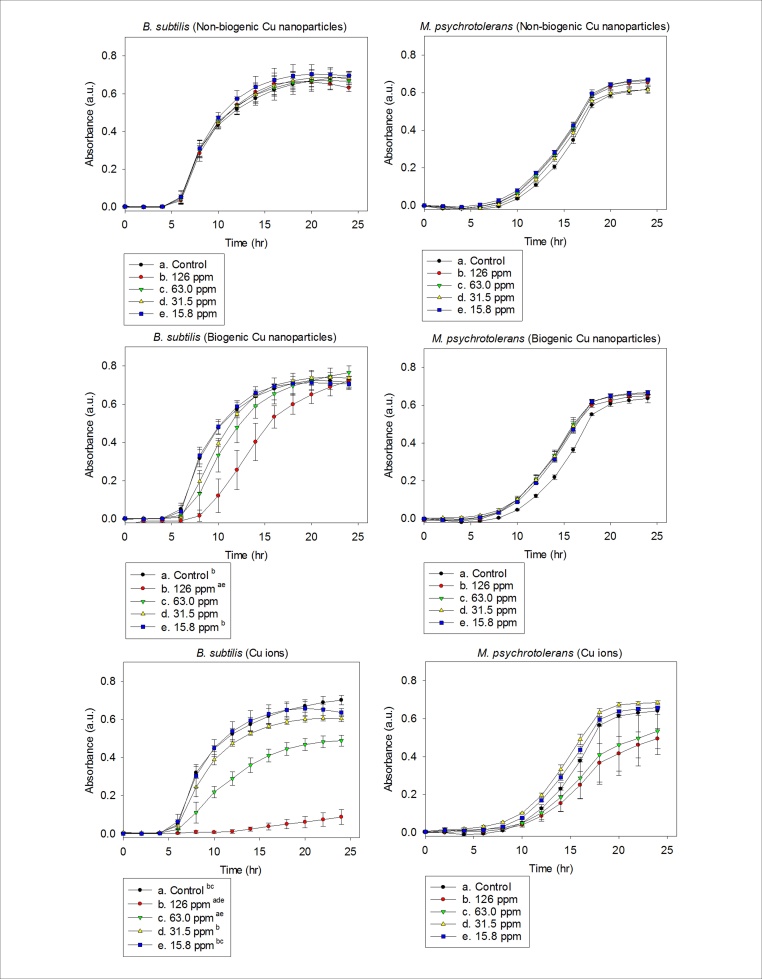
Graphs depicting growth of *B. subtilis* (a–c) and *M. psychrotolerans* (d–f) in chemically synthesised copper nanoparticles, biogenic nanoparticles synthesised by *M. psychrotolerans* and Cu^2+^ ions. Letters in superscript name the sample to which the growth rate is significantly different (p < 0.05, One Way ANOVA).

To study the anti-microbial effects of biogenic nanoparticles compared to copper ions, the stirred cell method was used to purify copper nanoparticles synthesised by *M. psychrotolerans*. These biogenic copper nanoparticles were compared to copper ions and chemically-derived commercially available copper nanoparticles for their anti-microbial activity on *Bacillus subtilis* and *M. psychrotolerans*. Copper nanoparticles from *M. psychrotolerans* showed an inhibitory effect on the growth of *B. subtilis* at concentrations of 126 ppm (Fig. 6b). Conversely, no inhibitory effect was observed using the commercially available nanoparticles (Fig. 6a). Copper ions at similar concentrations showed much more pronounced inhibitory effects and were used as the benchmark for comparison (Fig. 6c). The use of copper ions as anti-microbials is limited due to their long-term instability and restricted use to aqueous solutions. No significant inhibitory effect was seen on the growth of *M. psychrotolerans* even at the highest concentration tested for both biogenic and non-biogenic copper nanoparticles (Fig. 6d, e). Copper ions showed an inhibitory effect on *M. psychrotolerans* at concentrations of 126 and 63.0 ppm (Fig. 6f); however this was not lethal.

## Conclusion

Metallic nanoparticles are set to become a common feature of industrial and medical processes. Biogenic nanoparticle production methods such as described here offer a green, resource-efficient way of meeting the demand for nanoparticles. These biological systems can be engineered to further adapt them to industrial use and this work serves as a foundation to allow us to manipulate nanoparticle morphologies; we have shown here how manipulating pH can result in changes to particle morphology, including the ability to produce uniform, non-aggregated nanoparticles. Further understanding of the mechanisms behind copper nanoparticle formation will lead to much finer control over nanoparticle production and morphology. We have also shown that the copper nanoparticles produced using this method display inhibitory effects on the growth of a Gram-positive bacterium, indicating their potential as anti-microbial agents. By harnessing the natural ability of bacteria to produce useful nanoparticles, and improving it through engineering, we have the potential to move to large scale biomanufacture, controlling nanoparticle synthesis in order to achieve the desired morphologies, compositions and activities.

## Funding

This research was supported by the BBSRC and Diageo [BB/F017073/1], BBSRC [BB/N002520/1] and EPSRC [EP/K026216/1].
