# Supplementary material for: Room temperature bioproduction, isolation and anti-microbial properties of stable elemental copper nanoparticles
Source: N Biotechnol. 2018 Jan 25;40(Pt B):275–81. doi: 10.1016/j.nbt.2017.10.002 (PMC5734607; doi:10.1016/j.nbt.2017.10.002)
Supplement: Supplementary file 1 [file mmc1.docx]

Suplpementary Data

X-ray Photoelectron Spectroscopy (XPS) was performed on copper nanoparticles prepared over a period of six months to analyse their stability. The nanoparticles were stable and were observed to stay elemental in nature up until 3 months post-production. Thereafter the nanoparticles appeared to have oxidised based on XPS data collected (Figure A1). Presence of Cu_2_O was observed after 3 months of storage at 4^o^C.


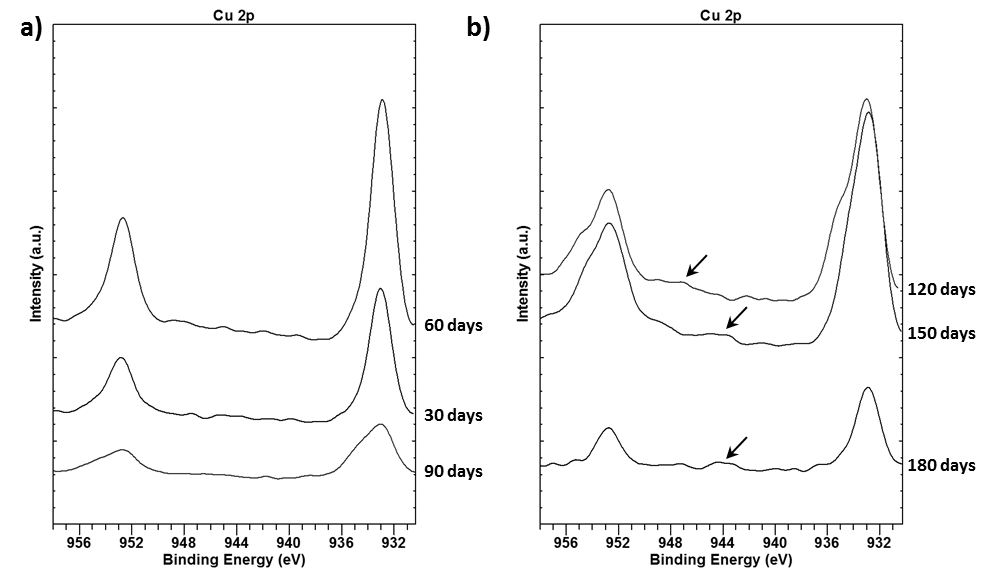


**Figure A1**. a) XPS spectrum showing the characteristic two peaks caused by the presence of elemental copper. b) A small satellite peak (black arrows) between the Cu 2p 1/2 (left peak) and Cu 2p 3/2 (right peak) peaks is indicative of the presence of Cu_2_O.
